# Differential patterns of sweat and blood lactate concentration response during incremental exercise in varied ambient temperatures: A pilot study

**DOI:** 10.1080/23328940.2024.2375693

**Published:** 2024-07-30

**Authors:** Naoya Takei, Takeru Inaba, Yuki Morita, Katsuyuki Kakinoki, Hideo Hatta, Yu Kitaoka

**Affiliations:** a Research Institute of Physical Fitness, Japan Women's College of Physical Education, Tokyo, Japan; b Department of Sports Sciences, The University of Tokyo, Tokyo, Japan; c BlueWych LLC, Atsugi, Kanagawa, Japan; d Department of Human Sciences, Kanagawa University, Yokohama, Kanagawa, Japan

**Keywords:** Blood lactate, sweat lactate, lactate threshold, energy metabolism, thermoregulation

## Abstract

Blood lactate concentration during exercise is a reliable indicator of energy metabolism and endurance performance. Lactate is also present in sweat, and sweating plays an important role in thermoregulation, especially in hot conditions. Recently, wearable sensors have enabled the real-time and noninvasive measurement of sweat lactate concentration, potentially serving as an alternative indicator of blood lactate response. However, the evidence regarding the relationship between sweat and blood lactate responses during incremental exercise in hot conditions is lacking. In a randomized cross-over design, six highly trained male runners completed two incremental treadmill tests under normal (20°C/50%RH) or hot (30°C/50%RH) conditions. The tests include 3-min running stages and 1-min recovery, starting at 12 km/h and increasing by 1 km/h at each stage. Blood and sweat lactate concentrations were measured at each stage to determine blood and sweat lactate thresholds (LT). Blood lactate concentrations were higher under hot conditions (*p* < 0.01), but there was no difference in the response pattern or velocity at blood LT between conditions. Significant early increase (*p* < 0.01) in sweat lactate and low velocity at sweat LT (*p* < 0.05) were observed under hot conditions. A significant correlation between blood and sweat lactate concentrations was found under normal conditions (*p* < 0.001) but not under hot conditions, and no significant correlations were observed between the velocity at blood and sweat LT. In conclusion, sweat lactate concentration does not consistently reflect blood lactate concentration during incremental exercise.

## Introduction

The measurement of blood lactate concentration is a common method for assessing energy metabolism during exercise. Lactate, a major glycolytic metabolite, acts as a key indicator of increased glycolysis in working muscles during exercise [1,2,3]. When measuring blood lactate concentration during incremental exercise, there is a specific point where blood lactate concentration rises rapidly at a given exercise intensity, known as the “lactate threshold” [4]. This threshold is widely recognized and utilized as a reliable indicator for assessing endurance exercise performance and prescribing training [2,5,6].

Sweating plays a critical role in thermoregulation during exercise, particularly in hot environments [7]. Sweat is composed of various substances such as electrolytes and also contains lactate [8]. Traditional methods to measure sweat lactate levels include whole-body washdown, plastic bags, drape-based collection, and absorbent patches [9–12]. However, these methods may influence the results and lack real-time measurement capabilities [13]. Recent advancements in wearable devices for sweat lactate measurement devices utilizing microfluidic-based sweat sampling methods and electrochemical sensors enable real-time monitoring of sweat lactate concentration during exercise [13–15]. Sweat which contains a large amount of lactate, is primarily derived from the metabolic activity of sweat glands [16,17]. Recent studies employing real-time sweat lactate sensors have indicated that the changes in sweat lactate concentration are consistent with those in blood lactate concentration during exercise [18,19]. Therefore, unlike invasive blood sampling for measuring blood lactate concentration, assessing sweat lactate concentration may provide a noninvasive method to evaluate energy metabolism during exercise.

Previous studies have shown a relationship between blood and sweat lactate responses during incremental or constant-load exercises in various populations, including healthy subjects, recreationally active subjects, or patients with cardiovascular disease [18–21]. This suggests that sweat lactate sensors can serve as viable alternatives to assess blood lactate responses. However, while sweat gland activity producing sweat lactate may be more pronounced in hot environments, previous studies have primarily focused on exercise in normal conditions rather than in hot environments. Furthermore, despite the widespread use of lactate threshold to evaluate endurance performance in athletes, no study has investigated sweat lactate responses during exercise in highly trained subjects.

Glycolysis is essential for the activity of sweat glands, particularly in eccrine glands [16,17]. The tracer study using 14C-labeled lactate or glucose demonstrated that sweat lactate primarily originates from the metabolism of blood glucose by sweat glands, rather than from blood lactate [16]. Consequently, variations in ambient temperature are likely to have a significant impact on sweat lactate response. Therefore, this study aimed to confirm the hypothesis that the sweat lactate response differs between normal (20°C) and hot (30°C) environments in highly trained runners. We expect that the increase in sweat lactate concentration (sweat lactate threshold) will occur significantly earlier in a hot environment (30°C) compared to a normal environment (20°C), while the timing of the increase in blood lactate concentrations (blood lactate threshold) will remain unchanged.

## Materials and methods

Six male endurance runners participated in this study after providing written informed consent. They trained at least five days a week, achieving personal best records within 20% of world-leading performance, which categorizes them as “Highly Trained” (Tier 3), according to the established criteria [22]. *Post hoc* power analysis using G*Power 3.1.9.7 (Heinrich-Heine-Universität Düsseldorf, Germany) confirmed high statistical power (1-*β* = 0.99, *α* = 0.05), based on the mean effect size (*d* = 2.74) for differences in the velocity at the sweat lactate threshold between the two experimental conditions (20°C *vs*. 30°C). The study was conducted following the Declaration of Helsinki and approved by the Research Ethics Committee of the University of Tokyo (No. 689–3).

This study employed a randomized crossover design. Participants performed incremental treadmill tests during separate visits, either 20°C/50% RH or 30°C/50% RH. The incremental treadmill tests comprised a 3-min running stage followed by a 1-min passive recovery period in a standing position, with reference to a previous study [23]. After participants performed self-conducted warm-up exercises (e.g. stretching and jogging), the incremental treadmill test began at a running speed of 12 km/h. The running speed was then increased by 1 km/h as each stage progressed using a motorized treadmill (T.K.K 1255, Takei Scientific Instrument, Japan). During the 1-min recovery period, blood lactate concentration was assessed using a portable lactate analyzer (Lactate Pro 2, Arkray, Japan) with blood samples obtained from a fingertip. This portable lactate analyzer has high reliability (coefficient of variation = 3.3%) compared to a reliable laboratory-based analyzer [24]. The incremental treadmill tests were terminated when blood lactate concentration exceeded 4 mmol/L. The blood lactate threshold (LT) was determined using the log-log transformation method [25]. Participants consumed a light meal, three hours before each visit and were instructed to replicate this meal as closely as possible. Participants were also directed to avoid consuming ergogenic-aid substances (e.g. caffeine and creatine) and engaging in heavy exercise 48 hours before the tests. All tests were spaced 5–7 days apart and conducted at the same time of the day to mitigate circadian effects.

Sweat lactate concentration was continuously monitored using a wearable sweat lactate sensor (Grace Imaging, Japan), as previously described [19]. The sensor was applied to the right upper arm after cleaning the installation area with an alcohol swab to prevent contamination. It was secured using a medical-grade waterproof film and surgical tape. Sweat lactate levels were measured using a dedicated device (Grace Imaging, Japan) via Bluetooth at a sampling rate of 1 Hz. Sweat lactate values were averaged over the last 30-s for each 3-min running stage. The Sweat LT was defined as the first significant increase in sweat lactate concentration above the baseline, determined graphically as described in previous studies [19–21].

Statistical analyses were conducted using Prism (v10.2.2; GraphPad Software, USA). Normality was assessed using the Shapiro-Wilk test. If normality assumptions are violated, the Wilcoxon signed-rank test was applied. A two-way repeated measures ANOVA [velocity (12, 13, 14, 15, 16, 17, 18, 19 km/h) × temperature (20°C, 30°C)] was performed to compare the dependent variables. Tukey post-hoc analysis was employed for multiple comparisons. Mauchly’s test of sphericity was employed for all ANOVA results. If these assumptions were violated, a Greenhouse-Geisser correction was applied to adjust the degrees of freedom. Pearson’s correlation analysis was used to examine the relationships between variables. All values are presented as the mean ± standard deviation, and statistical significance was set at *p* < 0.05.

## Results

**Figure 1.**
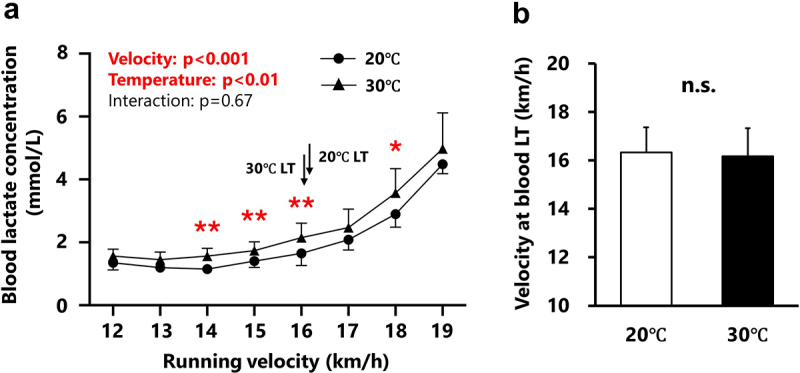
Changes in blood lactate concentrations during the test (Panel a) and velocity at the blood lactate threshold between conditions (Panel b).

Changes in blood lactate concentrations during the incremental treadmill test are depicted in Figure 1. Blood lactate concentration significantly increased (main effect of velocity, *p* < 0.001) with increasing velocity (Figure 1a). Under hot conditions (30°C), blood lactate concentrations were significantly higher (main effect of temperature: *p* < 0.01) compared to normal conditions (20°C) at 14, 15, 16, and 18 km/h (Figure 1a). The velocity at the blood lactate threshold did not differ between normal and hot conditions (16.2 ± 0.5 and 16.1 ± 0.6 km/h, respectively; Figure 1b).

Changes in blood lactate concentration during the incremental treadmill test under normal (circles) and hot (triangles) conditions are shown in Panel A. Panel B represents the velocity at the blood lactate threshold (LT) under normal (white bar) and hot (black bar) conditions. Arrows indicate the position of LT. All values are expressed as mean ± SD. *p < 0.05, **p < 0.01 between conditions. The n.s. indicates “not significant” between conditions.

**Figure 2.**
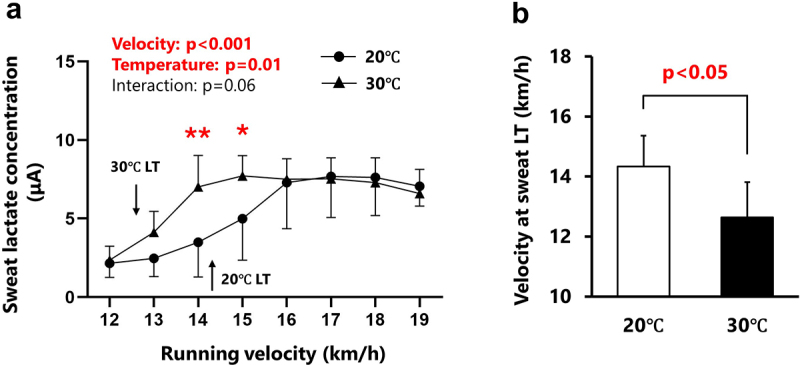
Changes in sweat lactate concentrations during the test (Panel a) and velocity at the sweat lactate threshold between conditions (Panel b).

**Figure 3.**
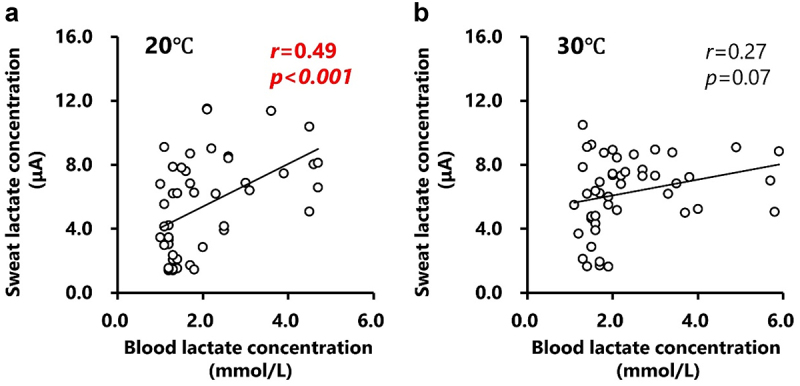
The relationship between sweat and blood lactate concentrations during the test in normal (Panel a) and hot conditions (Panel b).

**Figure 4.**
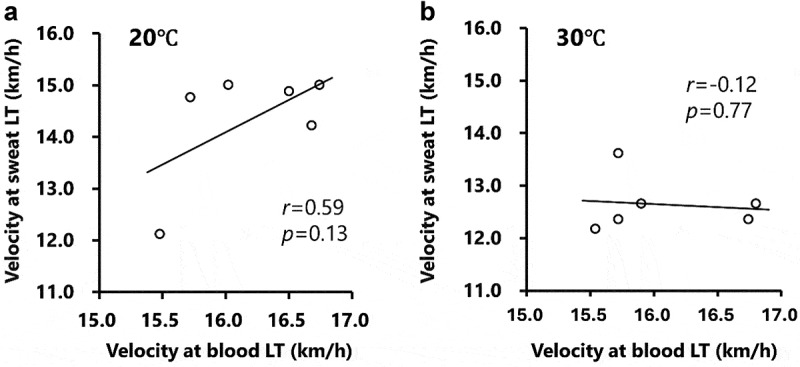
The relationship between sweat and blood lactate threshold during the test in normal (Panel a) and hot conditions (Panel b).

The changes in sweat lactate concentrations during the incremental treadmill test are shown in Figure 2. Sweat lactate concentration significantly increased (main effect of velocity: *p* < 0.001) with increasing velocity (Figure 2a). Under hot conditions (30°C), sweat lactate concentrations were significantly higher (main effect of temperature: *p* < 0.01) than under normal conditions (20°C) at 14 and 15 km/h (Figure 2a). A significant interaction effect was observed for sweat lactate responses, indicating different responses between conditions. The velocity at the sweat lactate threshold was significantly lower under hot conditions (12.6 ± 0.5 km/h; *p* < 0.05) than under normal conditions (14.3 ± 0.6 km/h; Figure 2b). Blood and sweat lactate concentrations at all time points correlated significantly under normal conditions (*r* = 0.49, *p* < 0.001; Figure 3a), but not under hot conditions (*r* = 0.27, *p* = 0.07; Figure 3b). The velocities at blood and sweat LT did not correlate in either the hot (*r* = −0.12, *p* = 0.77) or normal (*r* = 0.59, *p* = 0.13) conditions (Figure 4).

Changes in sweat lactate concentration during the incremental treadmill test under normal (circles) and hot (triangles) conditions are shown in Panel A. Panel B represents the velocity at the sweat lactate threshold (LT) under normal (white bar) and hot (black bar) conditions. Arrows indicate the position of LT. Sweat lactate concentration was increased significantly earlier in hot condition compared to normal condition. All values are expressed as mean ± SD. *p < 0.05, **p < 0.01 between conditions.

The relationship between sweat and blood lactate concentrations during the incremental treadmill test under normal (Panel a) and hot conditions (Panel b) is shown. Circle markers indicate the measured values and solid lines indicate the approximate straight lines for all values.

The relationship between sweat and blood lactate threshold (LT) during the incremental treadmill test under normal (Panel a) and hot conditions (Panel b) is shown. Circle markers indicate the measured values and solid lines indicate the approximate straight lines for all values.

## Discussion

One of the major findings of this study was the different response patterns of blood and sweat lactate during the incremental treadmill test in hot (30°C) and normal (20°C) conditions. Blood lactate concentrations were significantly higher at 14, 15, 16, and 18 km/h in hot conditions (Figure 1a). Consistent with these results, previous studies have also shown increased blood lactate concentration during exercise under hot conditions [26,27]. This phenomenon may be partly explained by a temperature-dependent increase in glycolytic enzyme activity [28]. Another potential explanation is that hot conditions may decrease cardiac output due to increased skin blood flow and/or loss of body water, leading to decreased blood flow to working muscles [7]. Reduced blood flow to the muscles can negatively affect exercise performance by increasing the relative intensity, thereby increasing blood lactate responses at a given running speed [7]. Despite the overall increase in blood lactate concentration under hot conditions, the velocity at blood LT did not differ between conditions. Around the velocity at blood LT, the accumulation of ADP and inorganic phosphate begins to increase, activating glycolysis [29]. Subsequently, glycolysis is enhanced to meet increasing energy demands. When lactate production in working muscle exceeds the rate of metabolism by mitochondria, lactate accumulates and is subsequently released into the blood stream by monocarboxylate transporters, resulting in a marked increase in blood lactate concentration (i.e. blood LT) [3]. Therefore, the identical velocity at blood LT between conditions (Figure 1b) implies that ambient temperature did not significantly affect the timing at which skeletal muscle energy demand increased. Contrary to the response of blood lactate concentration, sweat lactate concentration significantly increased earlier (14–15 km/h) under hot conditions compared to normal conditions (Figure 2a). Moreover, the velocity at the sweat LT was significantly lower (−11.3 ± 8.3%) in the hot condition than in the normal condition (Figure 2b), suggesting that the high ambient temperature accelerates the timing of sweat lactate increase during incremental exercise. Therefore, the results supported the hypothesis that the sweat lactate response differs between normal (20°C) and hot (30°C) environments in highly trained runners.

The tracer study demonstrated that sweat lactate primarily originates from the metabolism of blood glucose by sweat glands, rather than from blood lactate [16]. Consequently, variations in ambient temperature are likely to have a significant impact on sweat lactate response. This notion was supported by the previous study investigating blood and sweat lactate responses during constant load (30 min at 40% VO_2_max) or interval exercises (15 × 1-min intervals at 80% VO_2_max) in the hot condition (~32°C) [30]. The findings revealed higher blood lactate concentrations during interval exercise compared to constant load exercise (~5 mmol/L vs 1.5 mmol/L), while sweat lactate concentrations remained consistent between conditions (~10 mmol/L) [30]. In addition, previous research on heat acclimation have shown a decrease in sweat lactate concentration during exercise after both exercise training interventions in a hot environment and warm bath interventions without exercise [31]. This suggests that the heat acclimation-induced decrease in sweat lactate concentration is not necessarily mediated by exercise intervention (i.e. skeletal muscle adaptation). Previous studies utilizing the same wearable sweat lactate sensor employed in this study reported that the timing of the increase in blood lactate (i.e. blood LT) aligns with that of the increase in sweat lactate (i.e. sweat LT) levels during incremental or constant-load exercise [19–21]. However, these studies were conducted under normal temperature conditions: one study reported a temperature of ~ 25°C/42%RH and the others did not specify precise temperature information but also did not involve hot conditions. Thus, while increased energy demand in skeletal muscles (i.e. blood LT) and increased sweat gland activity (i.e. sweat LT) may occur simultaneously, the metabolism of working muscles and sweat glands is not directly related. Indeed, the correlation between blood and sweat lactate concentrations was statistically significant under normal conditions (*r* = 0.49, *p* < 0.001; Figure 3a). Importantly, however, the velocity at sweat and blood LT did not significantly correlate under either normal (*r* = 0.59 and *p* = 0.13) or hot (*r* = −0.12, *p* = 0.77) conditions (Figure 4). Given that sweat lactate is derived from the metabolic activity of sweat glands [16,17] but not from the blood lactate, sweat lactate concentration cannot always serve as an alternative indicator of blood lactate, especially under hot conditions.

One possible limitation was the small and homogeneous sample size (*n* = 6; only highly trained athletes) of this pilot study. The small sample size may lead to an underpowered analysis of the relationships between blood and sweat lactate responses, although a medium effect size (*r* = 0.49) was observed in the normal condition [32]. Additionally, due to the small sample size, correlative data may be affected by variations in the responses between individuals. Further studies with larger sample sizes, including subjects in various training status, are needed to address this limitation.

In conclusion, blood and sweat lactate concentrations exhibit different kinetics during the incremental treadmill test in highly trained runners in normal (20°C/50%RH) or hot environments (30°C/50%RH). Blood lactate concentrations were higher overall in hot conditions, whereas sweat lactate showed an earlier increase compared to normal conditions. Sweat lactate concentration is not always a reliable indicator of the blood lactate response, especially under hot conditions.

## Abbreviations

ANOVA: Analysis of variance
ADP: Adenosine diphosphate
14C: Carbon-14
LT: Lactate threshold
RH: Relative humidity
VO_2_max: Maximal oxygen consumption
